# Fiducial detection and registration for 3D IMRT QA with organ‐specific dose information

**DOI:** 10.1002/acm2.13237

**Published:** 2021-03-31

**Authors:** Yi‐Fang Wang, Olga Dona, Yuanguang Xu, John Adamovics, Cheng‐Shie Wuu

**Affiliations:** ^1^ New York‐Presbyterian/Columbia University Irving Medical Center (NYP/CUIMC) New York NY USA; ^2^ Rider University Lawrenceville NJ USA

**Keywords:** 3D IMRT QA

## Abstract

**Purpose:**

Two‐dimensional (2D) IMRT QA has been widely performed in Radiation Oncology clinic. However, concerns regarding its sensitivity in detecting delivery errors and its clinical meaning have been raised in publications. In this study, a robust methodology of three‐dimensional (3D) IMRT QA using fiducial registration and structure‐mapping was proposed to acquire organ‐specific dose information.

**Methods:**

Computed tomography (CT) markers were placed on the PRESAGE dosimeter as fiducials before CT simulation. Subsequently, the images were transferred to the treatment planning system to create a verification plan for the examined treatment plan. Patient’s CT images were registered to the CT images of the dosimeter for structure mapping according to the positions of the fiducials. After irradiation, the 3D dose distribution was read‐out by an optical‐CT (OCT) scanner with fiducials shown on the OCT dose images. An automatic localization algorithm was developed in MATLAB to register the markers in the OCT images to those in the CT images of the dosimeter. SlicerRT was used to show and analyze the results. Fiducial registration error was acquired by measuring the discrepancies in 20 fiducial registrations, and thus the fiducial localization error and target registration error (TRE) was estimated.

**Results:**

Dosimetry comparison between the calculated and measured dose distribution in various forms were presented, including 2D isodose lines comparison, 3D isodose surfaces with patient’s anatomical structures, 2D and 3D gamma index, dose volume histogram and 3D view of gamma failing points. From the analysis of 20 fiducial registrations, fiducial registration error was measured to be 0.62 mm and fiducial localization error was calculated to be 0.44 mm. Target registration uncertainty of the proposed methodology was estimated to be within 0.3 mm in the area of dose measurement.

**Conclusions:**

This study proposed a robust methodology of 3D measurement‐based IMRT QA for organ‐specific dose comparison and demonstrated its clinical feasibility.

## 1 INTRODUCTION

Intensity modulated radiation therapy (IMRT) quality assurance (QA) or patient‐specific QA has been widely performed in the clinic to verify treatment planning dose calculation as well as the delivery system of a linear accelerator (LINAC) with multileaf collimators (MLCs).^1^, ^2^, ^3^, ^4^ However, its sensitivity in detecting errors and its relevance to clinical judgment has been extensively discussed by physicists.^5^, ^6^, ^7^, ^8^ In 2018, AAPM task group 218 was published in order to address the issues of IMRT QA and review the existing measurement‐based methods and computer reconstruction methods.^9^ It was concluded that the conventional gamma test should be reviewed on a structure by structure basis if the QA method allows for it. Purely using the passing rate for evaluation could underestimate the clinical consequences because the passing rate only summarizes the gamma test in aggregate. In addition, computed and measured DVH comparisons can provide more clinically relevant information. The study also addressed that the dose difference criterion would ideally be customized for different anatomical structures and the predicted dose in the structures. For example, the dose criterion in the spinal cord for a predicted cord dose of 45 Gy should be tighter than the tolerance in the cord with a predicted dose of 20 Gy. A recent study evaluated current measurement‐based QA at multiple institutions using the IROC head and neck IMRT phantom.^10^ The results showed that traditional IMRT QA methods performed consistently poorly in searching for a large error or a moderate error regardless of whether a 3%/3 mm or a 2%/2 mm criteria was used.

This work aims to resolve the issues regarding IMRT QA by demonstrating a measurement‐based methodology using fiducial registration and structure‐mapping to acquire organ‐specific dose information. PRESAGE three‐dimensional (3D) dosimeters (Heuris Inc.) have been recognized as true 3D dosimeters because dose deposition in the 3D space is readout using an optical scanner with no computer modeling involved.^11^, ^12^ The dosimeter consists of an optically clear polyurethane matrix, containing a leuco dye and free radical initiators that exhibits a radiochromic response when exposed to ionizing radiation. In 2012, the first comprehensive application of 3D dosimetry to verify a complex radiation treatment was proposed.^13^ The novelty of this work was to transform measured 3D dose distribution in the phantom back to the patient CT data, and thus enabling DVHs comparison. However, the study addressed that the methodology was limited to the accuracy of the 3D dose measurement, as well as the dose transformation between the phantom CT and the patient CT since the dose deposition at the two different geometries cannot be adequately described by a simple transformation matrix. Also, it was not clear how the correlation between the coordinates of the evaluation space and the reference space was established.

Furthermore, several publications have shown 3D dose measurement of IMRT fields using different types of 3D dosimeters (Gel, PRESAGE etc) and dose read‐out tools.^11^, ^23^ One of the most significant source of errors remains in the 3D registration between the measured dose and planned dose, which requires fiducial markers to be shown with sufficient contrast in two different image modalities, simulation CT and optical CT. The registration error is important for 3D measurement‐based QA because the dosimeters were read‐out by an optical CT scanner with different orientations than the CT scan. No previous research has analyzed the effect of the registration errors in 3D dose comparison, or have reported the accuracy of the registration. Previous studies have addressed that the result of registration errors in the manual alignment of the measured and calculated dose distributions leads to the gamma failing points at the sharp dose gradient regions.^15^, ^18^ A robust and accurate registration between the treatment planning coordinates and the dosimeter coordinates is therefore one of the key components to true 3D dose comparison. One could find the ‘best match’ through the extended use of manual registration. However, a rigorous and fair dose distribution comparison cannot be established when exclusive manual registration is used to align the dose distributions and the results are operator‐dependent. This study aims to resolve these concerns by proposing a methodology using automatic fiducial registration algorithm and commercially available structure‐mapping application in clinical TPS. First of all, fiducial‐based registration was employed to register the optical CT dose images to the simulation CT dosimeter images in order to correlate the two coordinate systems. Second, using the coordinates of the fiducials, patients’ anatomical structures were mapped to the dosimeter coordinates for structure‐by‐structure 3D dose comparison using Eclipse structure mapping application (Varian Inc). Finally, measured and calculated 3D dose distributions on the phantom were compared using clinically relevant information such as dose volume histogram (DVH), three‐dimensional (2D) dose distribution in any arbitrary plane and spatial positions of the failing gamma points in 3D. The main goal of this work is to propose a robust methodology of 3D measurement‐based IMRT QA with organ‐specific dose information and demonstrate its clinical feasibility. With the acquired information, organ‐specific dose difference criterion could be implemented in the future.

## 2 MATERIALS AND METHODS

### 2.A 3D IMRT QA with organ‐specific dose information

**Fig. 1:**
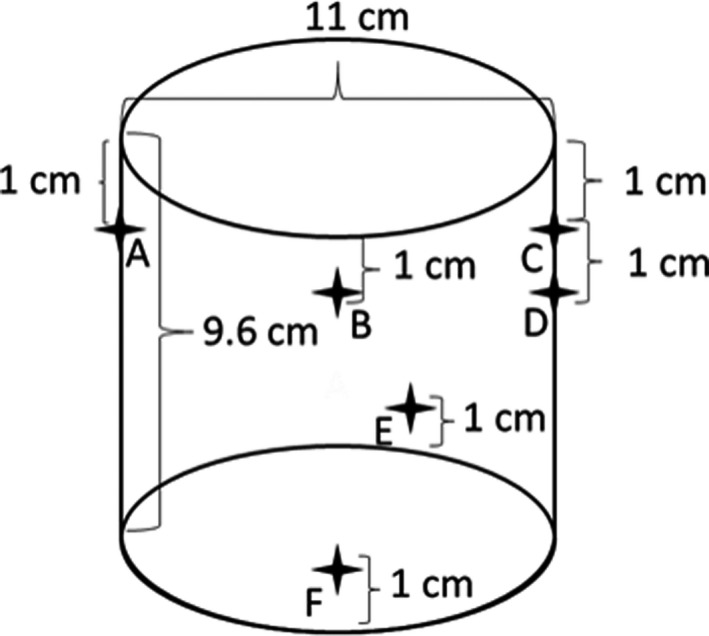
Diagram of a PRESAGE dosimeter with six fiducials placed on the surface.

**Fig. 2:**
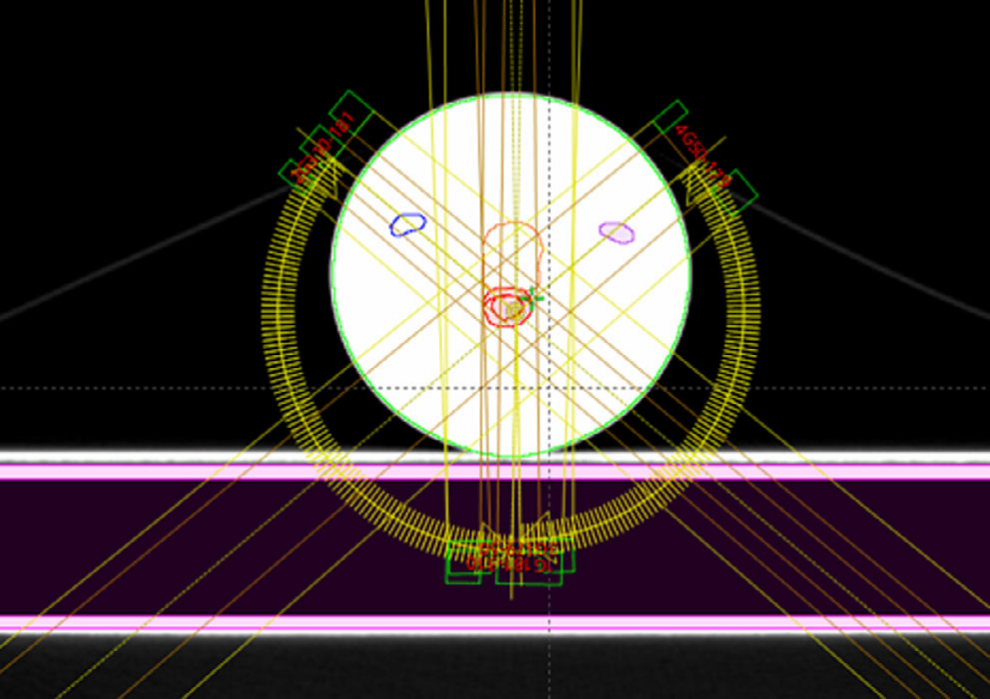
An axial view of a PRESAGE dosimeter placed on the treatment couch.

**Fig. 3:**
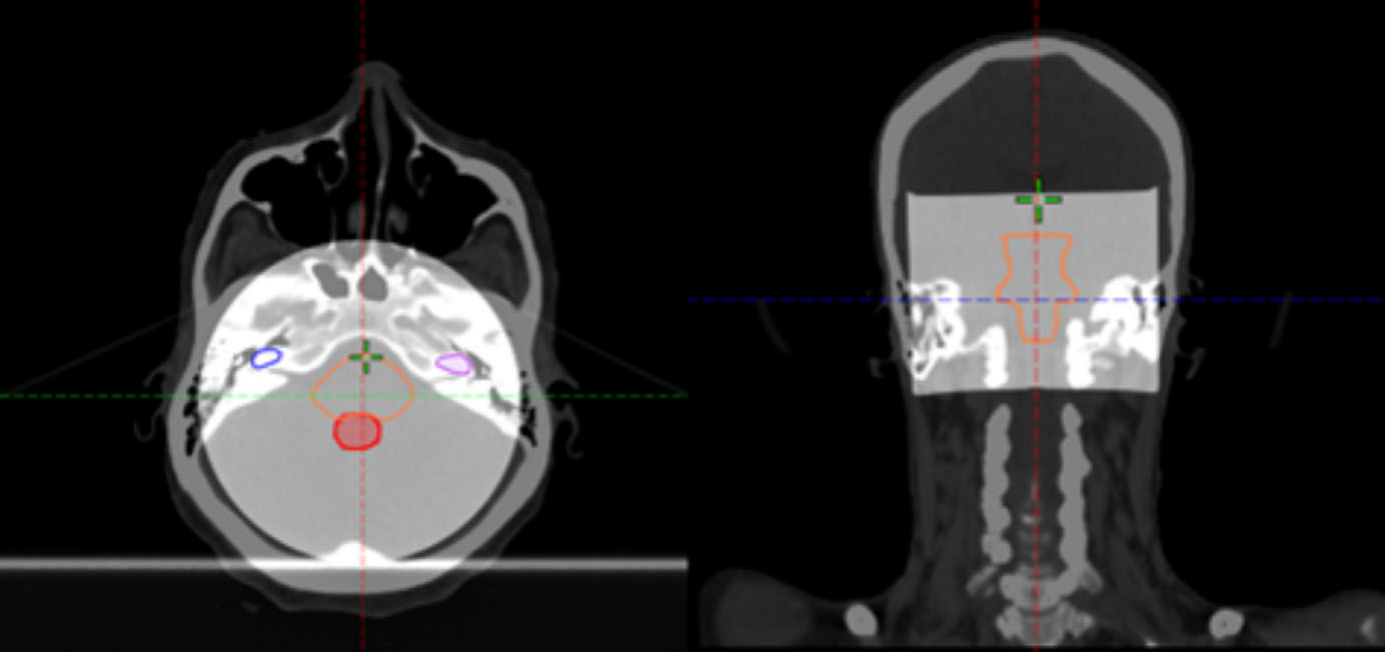
Mapping patient’s anatomical structures from patient’s CT image volume to phantom’s CT image volume. The two image volumes were registered based on the treatment plan isocenter. (Left: axial view, Right: coronal view).

The proposed methodology includes four main phases: CT simulation, dosimeter irradiation, dosimeter readout, and registration. In the first phase, six CT skin markers (Beekley Medical Inc.) were placed on the PRESAGE dosimeter with two purposes: setting up the dosimeter for the CT simulation and irradiation, and registration between the measured dose distribution and calculated dose distribution. Figure 1 shows the dimensions of the dosimeter and the relative positions of the six fiducials. Fiducial A, B, and C were aligned to the lasers before the CT simulation and treatment field irradiation. Fiducial D was used for left‐right discrimination when it was placed on the couch. Fiducial A, C, E, and F were employed as the registration markers. The geometrical positions of the fiducials were designed to achieve the optimal target registration errors, which were both calculated and measured in the study. As shown in Fig. 2, the dosimeter was placed on the couch with the axial plane perpendicular to the couch surface. A CT simulator, SOMATOM Definition AS (Sienmens, Inc) was used to acquire CT images of the dosimeter with 120 kVp and 1 mm slice thickness. In this study, two real patient plans were used as examples. The first case is a VMAT treatment of cerebellar metastasis (Fig. 2) with a total dose of 27 Gy in three fractions; the second case is a single isocenter, multiple lesions VMAT treatment of secondary malignant neoplasm of brain with 21 Gy in three fractions. The Acuros‐XB dose calculation algorithm (version 15.6, Varian, Inc) was used to calculate the dose distribution with a 1 mm calculation grid size, and hybrid plan verification of the VMAT treatment plans were created (Fig. 2) with the same dose grid size. A shift in the longitudinal direction was used to move the irradiation isocenter from the setup position to the central region of the dosimeter. With the setup fiducials, the setup position can be accurately identified. Using Eclipse image registration software, PRESAGE CT images were registered to the patient CT images based on the irradiation isocenter and then the anatomical structures (GTV, PTV, brain stem, chiasm, left cochlea and right cochlea) from the patient CT image volume were mapped to the registered PRESAGE CT images volume (Fig. 3). After the treatment plan preparation, the dosimeter was positioned in the treatment room for the irradiation of the verification plan using a Varian TrueBeam LINAC (Varian, Inc). In this study, the dosimeter received only one fraction of dose while in the Results section, the measured dose was scaled to the prescribed total dose for the presentation.

**Fig. 4:**
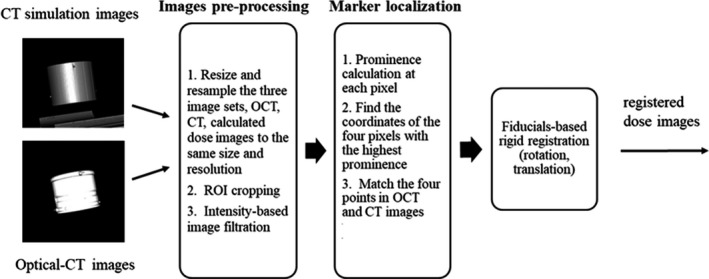
Schematic diagram of the developed algorithm for automatic fiducial detection and registration.

**Fig. 5:**
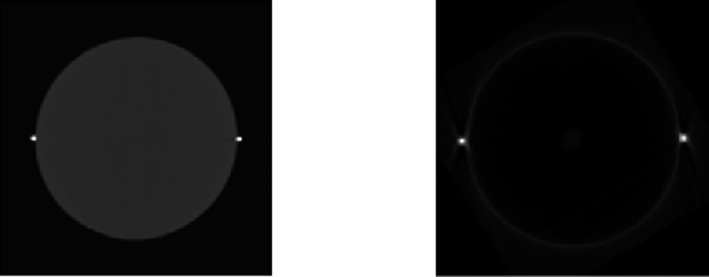
Registered CT simulation images (left) and OCT reconstructed images (right).

After irradiation, the 3D dose distribution of the irradiated dosimeter was readout by a single laser beam optical‐CT scanner (OCT) modified from the OCTOPUS^TM^ scanner^11^ at our institution. Four hundred projections were generated for one slice with slice thickness of 1 mm. For each projection (13.5 cm), 5000 data points were acquired. 3D dose images with submillimeter resolution were reconstructed using filtered back‐projection algorithm. An in‐house MATLAB (MathWorks, Inc) code was developed to perform the reconstruction algorithm and an automatic fiducial localization algorithm to register the markers in the OCT dose images to the CT simulation images. Figure 4 shows each step of the algorithm. Before localizing the markers, three image sets, CT simulation images, calculated dose images, OCT dose images were resampled to have the same size and resolution (1 mm). A region of interests (ROI) was selected to reduce the image size and pixels with image intensities higher or lower than a specific range were filtered out. In the marker localization phase, the prominence, of each pixel was calculated for both the CT and OCT image sets. The prominence measures how one pixel stands out from the surrounding pixels. Four pixels with the highest prominence values were selected in both image sets representing the fiducial points. Using singular‐value decomposition, rotation (R), and translation matrix (t) for the point‐based registration were found^24^:

(1) $$XY^{t}=U\Lambda V^{t}$$

(2) $$R=VDU^{t}$$

(3) $$t=\bar{y}‐R\bar{x},$$

where X, Y are the matrices, consisting of three rows and four columns. U,V are left and right singular vector matrices. The elements of each column in X and Y are the coordinates of the four fiducial points in the two image sets, respectively. D=diag(1,1,det(VU)), $\bar{x}$ and $\bar{y}$ are the first column in X and Y. By applying the rotation and translation matrix to the OCT images, the OCT images were registered to the CT images and the calculated dose images from TPS (Fig. 5). All the medical images, structures and dose images were imported to 3D Slicer, an open‐source software platform for image processing and visualization. SlicerRT, an extension of 3D Slicer, was employed in this study to visualize the structure sets, the measured and calculated dose distributions on the phantom as well as to calculate the DVHs, isodose lines, and gamma index.

### 2.B Registration error estimation

The overall dose comparison errors between calculated dose on the phantom and measured dose include real delivery errors to be detected, dosimetry uncertainties and registration errors between two different image modalities, CT and OCT dose images in 3D. Previous studies have examined the dosimetry uncertainties using 3D dosimeters and various read‐out techniques extensively^11^, ^23^ while the registration errors have not been analyzed in detail. In this study, fiducial localization error (FLE), which is the error in locating the fiducials, fiducial registration error (FRE), which is the root mean square distance between corresponding fiducials after registration, and target registration error (TRE), which is the distance between corresponding targets (not fiducials) after registration, were used to evaluate the fiducial registration technique. FRE was evaluated by comparing the coordinates of the fiducial points in the registered OCT images and the CT images. Twenty fiducials were registered to evaluate FRE. Using approximate expressions derived by Fitzpatrick et al., the expected squared FLE can be calculated from FRE^25^, ^26^:

(4) $$\langle FRE^{2}\rangle =\left(\left(N‐2\right)/N\right)\langle FLE^{2}\rangle$$

where N is the number of the fiducials. The expected squared FLE can be used in the following equation to predict the expected squared TRE at a point r:

(5) $$\langle TRE^{2}\left(r\right)\rangle \approx \frac{\langle FLE^{2}\rangle }{N}\left(1+\frac{1}{3}\sum_{k=1}^{3}\frac{d_{k}^{2}}{f_{k}^{2}}\right)$$

where $\langle FLE^{2}\rangle$ is the expected squared FLE, and N is the number of the fiducials. $d_{k}$ is the root‐mean‐square (RMS) distance to axis k for the evaluated point, r, and $f_{k}$ is the RMS distance to axis k for the fiducials. FLE and FRE relate to the image qualities of the OCT and CT images while TRE is influenced by the number and the location of the fiducials placed on the PRESAGE phantom. In this study, TRE was calculated and directly measured. To directly measure TRE, four fiducials were placed on the phantom as registration markers and ten fiducials were placed on the same phantom as the targets for evaluation.

## 3 RESULTS

### 3.A 3D IMRT QA with organ‐specific dose information

**Fig. 6:**
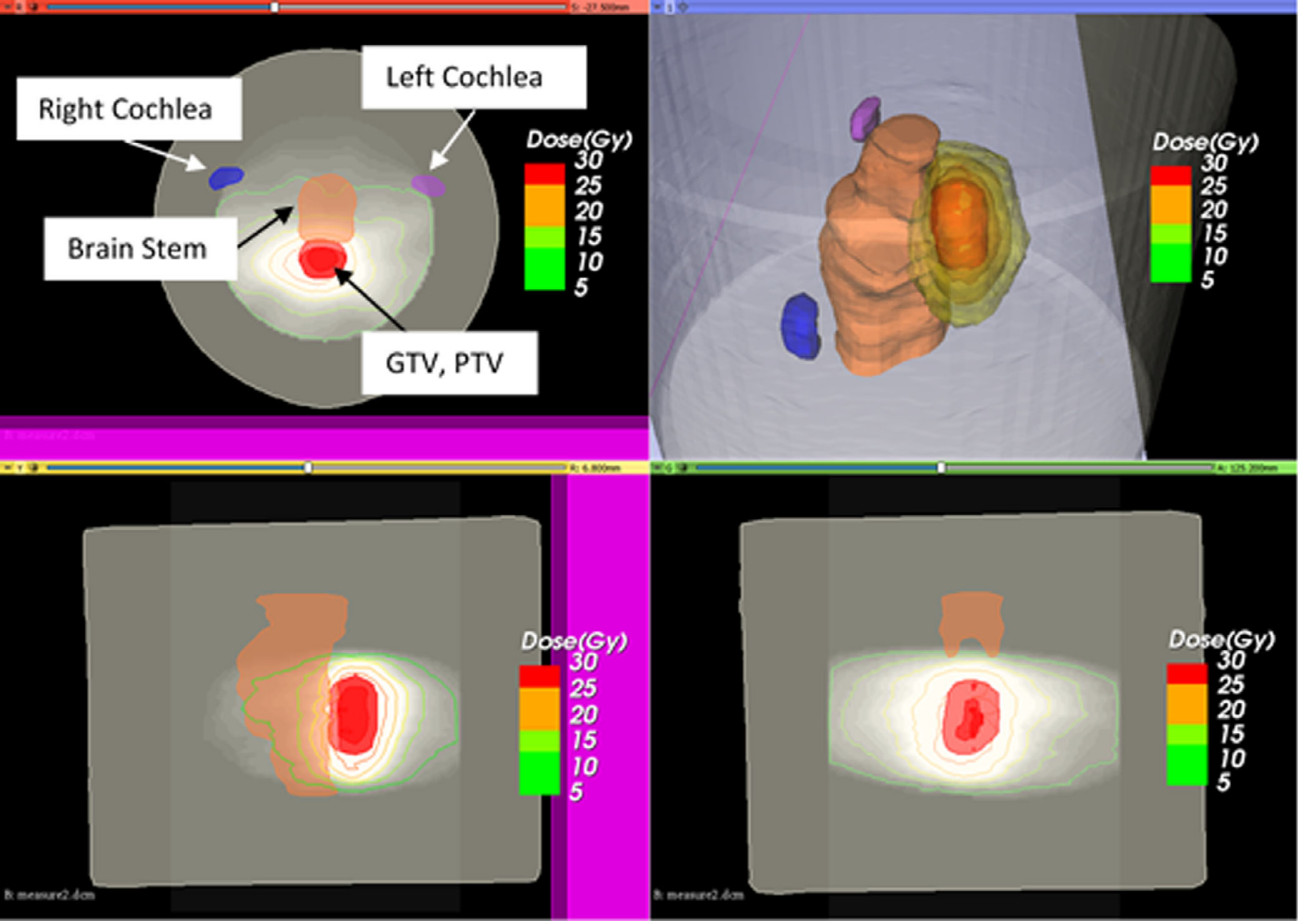
Imported three‐dimensional (3D) measured dose distribution of the cerebellar metastasis case and patient’s anatomical structures in 3D Slicer.

The proposed 3D IMRT QA method can provide relevant clinical information for the patient’s treatment plan, including evaluation of 2D isodose lines and gamma index at any arbitrary plane, 3D views of the dose distribution and structures, DVH of the targets and organs at risks (OAR), 3D gamma index and the location of the failing points relative to the structures. In the first case, a VMAT plan for cerebellar metastasis was selected for the demonstration. Figure 6 shows the results of the measured dose distribution and its relative location to the structures from 3D Slicer. In this case, the target is close to the brainstem and thus, sparing of the OAR is critical. With the proposed method, dose fall‐ off in the high dose gradient region between the target and the OAR can be evaluated.

**Fig. 7:**
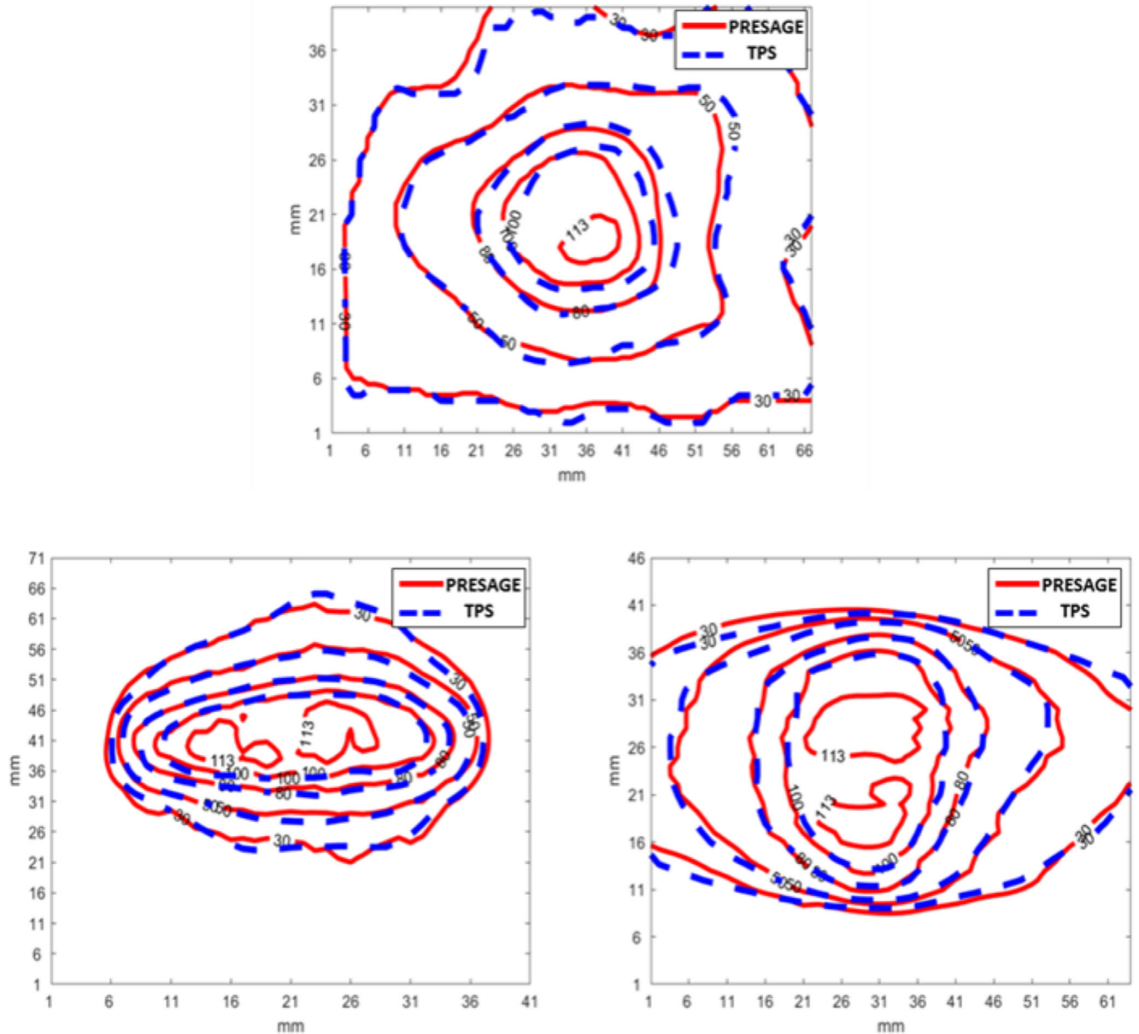
Three orthogonal views of the measured (red) and TPS‐calculated (blue) dose distribution comparison.

**Fig. 8:**
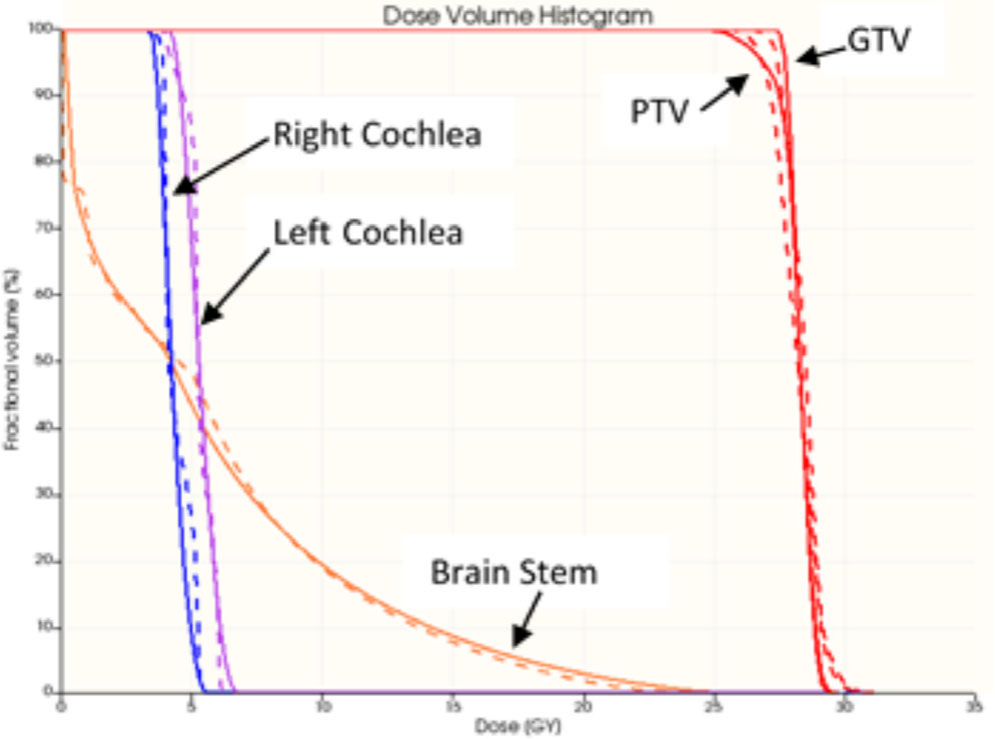
DVH comparison of the measurement (dashed line) and TPS‐calculation (solid line) of the cerebellar metastasis case.

In Fig. 7, isodose line comparison of the measured and calculated dose distribution on three orthogonal planes is presented. Two‐dimensional isodose comparison is a straightforward evaluation of the measured dose distribution. The presented case shows a good agreement between the measurement and the calculation at all dose levels except for the hot spots. The maximum doses of the calculated and measured dose distributions were 109% and 103%, respectively. Gamma analysis showed the passing rates of 99% for all three orthogonal planes respectively (using 3% and 3 mm criteria) and 97%, 98%, and 97% for the transverse, sagittal, and coronal planes (using 3% and 2 mm criteria). In addition to conventional 2D dose comparison, using the SlicerRT, we can calculate the DVHs and 3D gamma. Figure 8 presents the DVH comparison between the calculation and the measurement for this examined case. The measured coverage for the target is slightly lower than the calculated one. For the GTV and PTV, V27 Gy is 100% and 93.7% in the calculated dose distribution and 99.2% and 91.4% in the measured distribution. In addition, the hot spot value from the measurement (113%) is higher than what obtained by calculation (109%). For the brainstem, the measured mean dose and maximum dose were 5.5 and 24.7 Gy while the calculated doses were 5.5 and 24.3 Gy, respectively. The received maximum dose of the right and left cochlea are much smaller than the constraint (17 Gy) in this plan. The calculated maximum dose of the right and left cochlea were 5.49 and 6.75 Gy while the measured were 5.67 and 6.39 Gy.

**Fig. 9:**
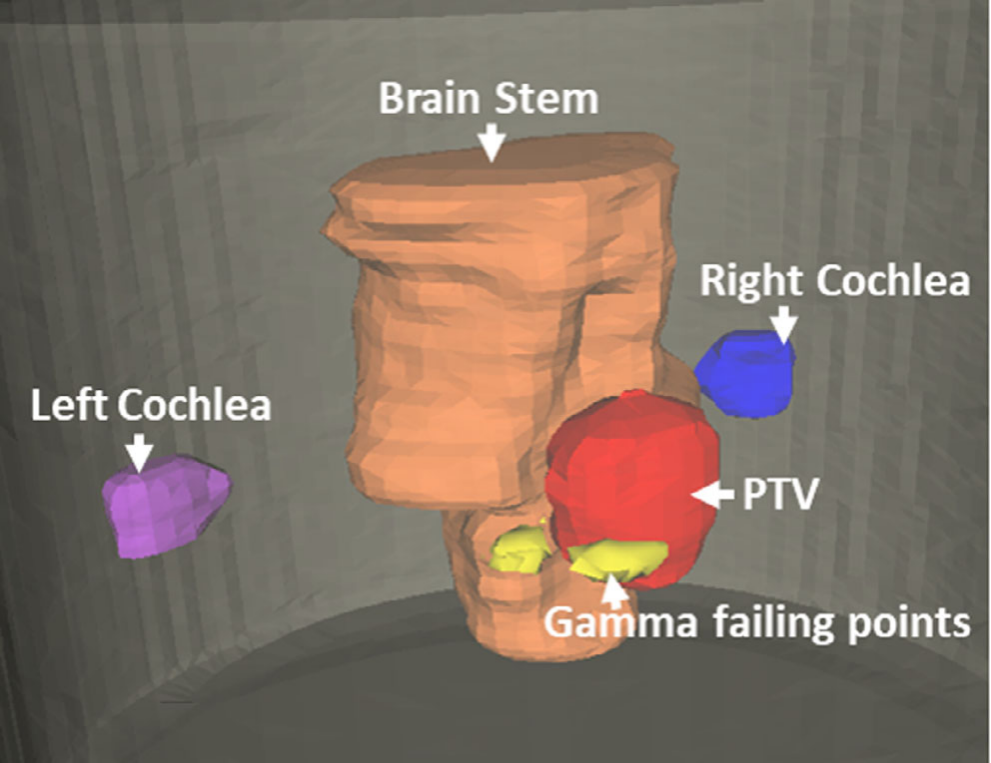
Visualization of the gamma failing points (yellow) in three‐dimensional space.

In addition to the DVH comparison, 3D gamma analysis was performed on the measured and calculated dose matrices. The passing rates were 99.2% and 96% using 3%, 3 mm and 3%, 2 mm criteria (with a 30% threshold). However, merely looking at the passing rate is challenging to make a clinical judgment. Using 3D Slicer, pixels that fail the 3%, 3 mm gamma test can be shown in 3D space (Fig. 9). The failing pixels are mostly in the region of a steep dose fall‐off outside the PTV, where the coverage of the PTV is influenced.

**Fig. 10:**
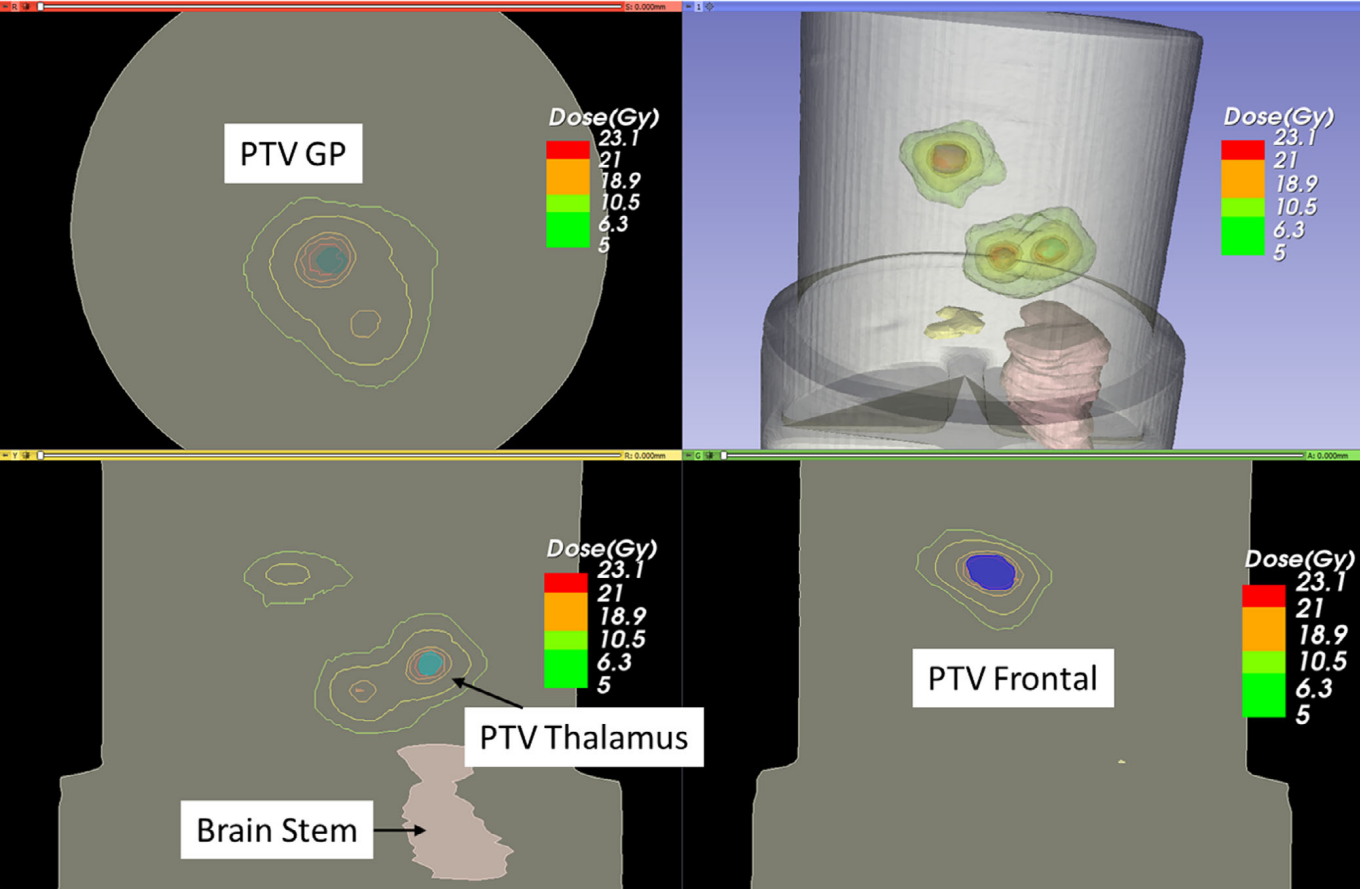
Imported three‐dimensional (3D) measured dose distribution of the multi‐lesions case and patient’s anatomical structures in 3D Slicer.

In the second case, three malignant lesions in brain, PTV at the frontal lobe (PTV frontal), PTV near thalamus (PTV thalamus) and PTV near globus pallidus (PTV GP) were irradiated using three non‐coplanar arcs with a single isocenter. Figure 10 shows the measured dose distribution in 3D using 3D Slicer. In this case, high gradient dose regions were scattered at different places to cover three targets. Both OAR, chiasm and brain stem were in the low dose region. The proposed method not only assessed the dose coverage of individual lesions but also the dose fall‐off outside the targets and low dose spill into normal brain.

**Fig. 11:**
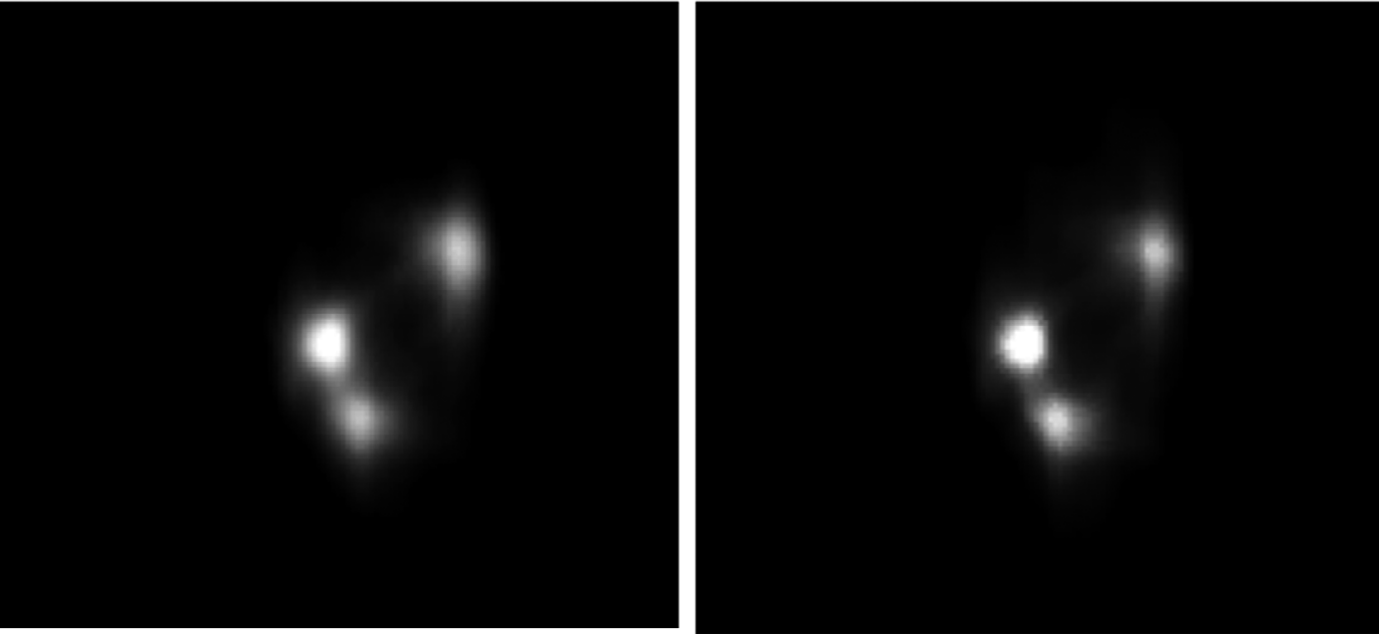
Extracted oblique slice images from measurement (left) and calculated (right) dose volume.

**Fig. 12:**
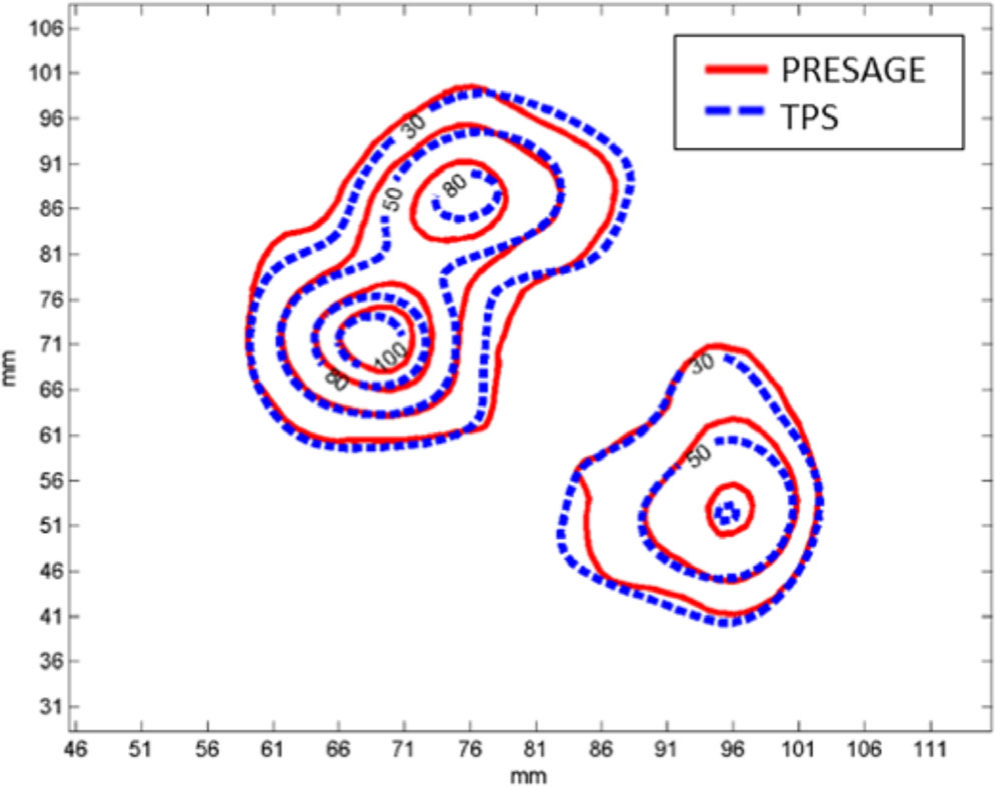
Measured (red) and TPS‐calculated (blue) dose distribution comparison of the extracted oblique plane.

**Fig. 13:**
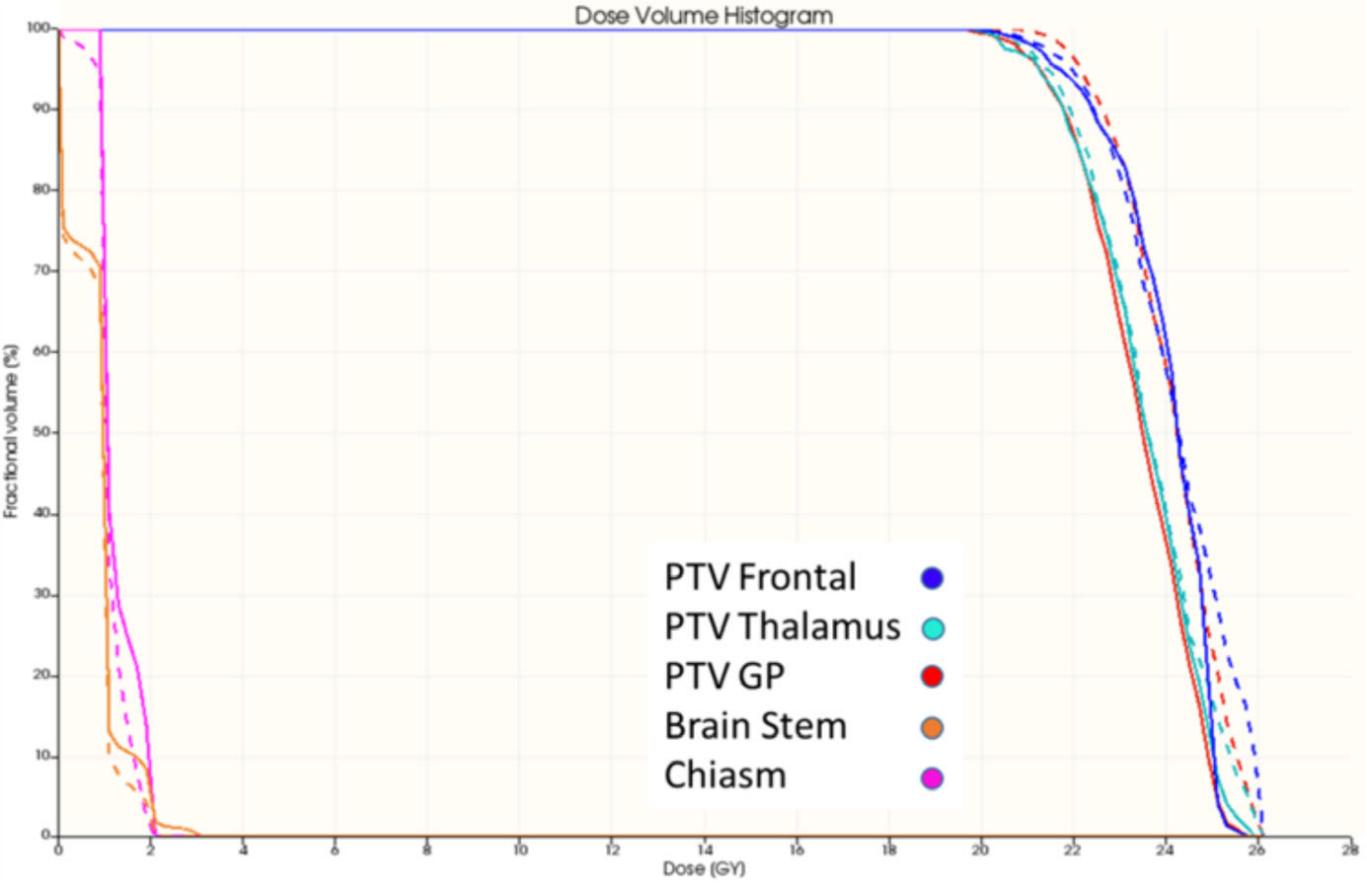
DVH comparison of the measurement (dashed line) and TPS‐calculation (solid line) of the multi‐lesions case.

An oblique slice showing three targets was extracted from the 3D measured dose volume and compared with the calculated dose. Figures 11 and 12 show the reconstructed image and planning image of the slice as well as their dose distribution comparison. The gamma passing rates of this slice are 96.2% and 91.6% using 3%/3 mm and 3%/2 mm criteria. The measured dose in region connecting the two close targets were higher than the calculated dose. Figure 13 presents the DVH comparison of the measured and calculated organ dose. The measurement shows that 95% of PTV Frontal, PTV Thalamus, and PTV GP receive at least 21.9, 21.3, and 22.1 Gy with maximum dose of 26 Gy while the calculation shows that 95% of PTV Frontal, PTV Thalamus, and PTV GP receive at least 21.1, 21.1, and 21.7 Gy with maximum dose of 25 Gy. For chiasm, the measured maximum dose is 2 Gy and calculated maximum dose is 2.25 Gy. Brain stem dose from calculation and measurement were both much lower than the constraint, 12 Gy. The 3D gamma passing rates were 98.03% and 91.52% using 3%, 3 mm and 3%, 2 mm criteria. Most of the gamma failing points are at the intermediate dose region (50–70%) between the two close lesions.

### 3.B Registration error estimation

**Fig. 14:**
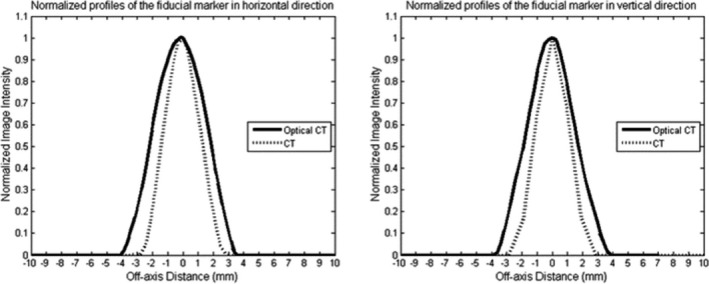
Normalized profiles of the fiducial markers on CT and OCT images in horizontal (left) and vertical (right) direction.

Essential factors affecting FLE and FRE are the image features of the fiducial markers in both image sets (OCT and CT images). In Fig. 14, normalized profiles of the fiducial markers are presented for both image sets. Due to different attenuation of the light sources (a HeNe laser and 120 kV photon beam), the shape of the fiducial markers on the images was different. The higher contrast of the fiducials in the CT images leads to narrower beam profiles of the fiducials in the CT images than the OCT images. Most importantly, in both image sets, one pixel of the peak value represents the location of the fiducials. This critical feature result in submillimeter FLE and FRE.

**Fig. 15:**
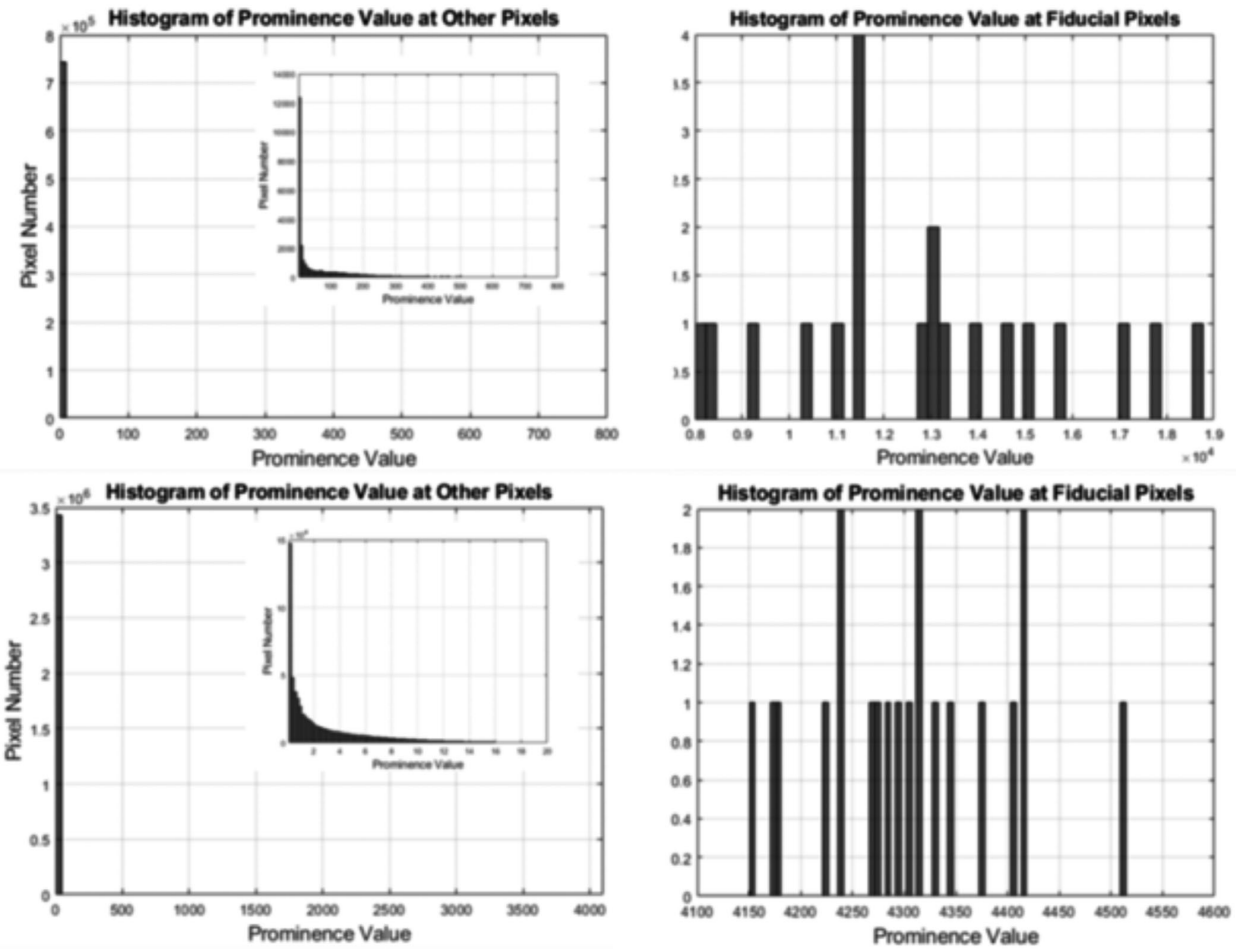
Prominence Values of the fiducial pixels (right) and other pixels (left) on CT (top) and OCT images (bottom).

**Fig. 16:**
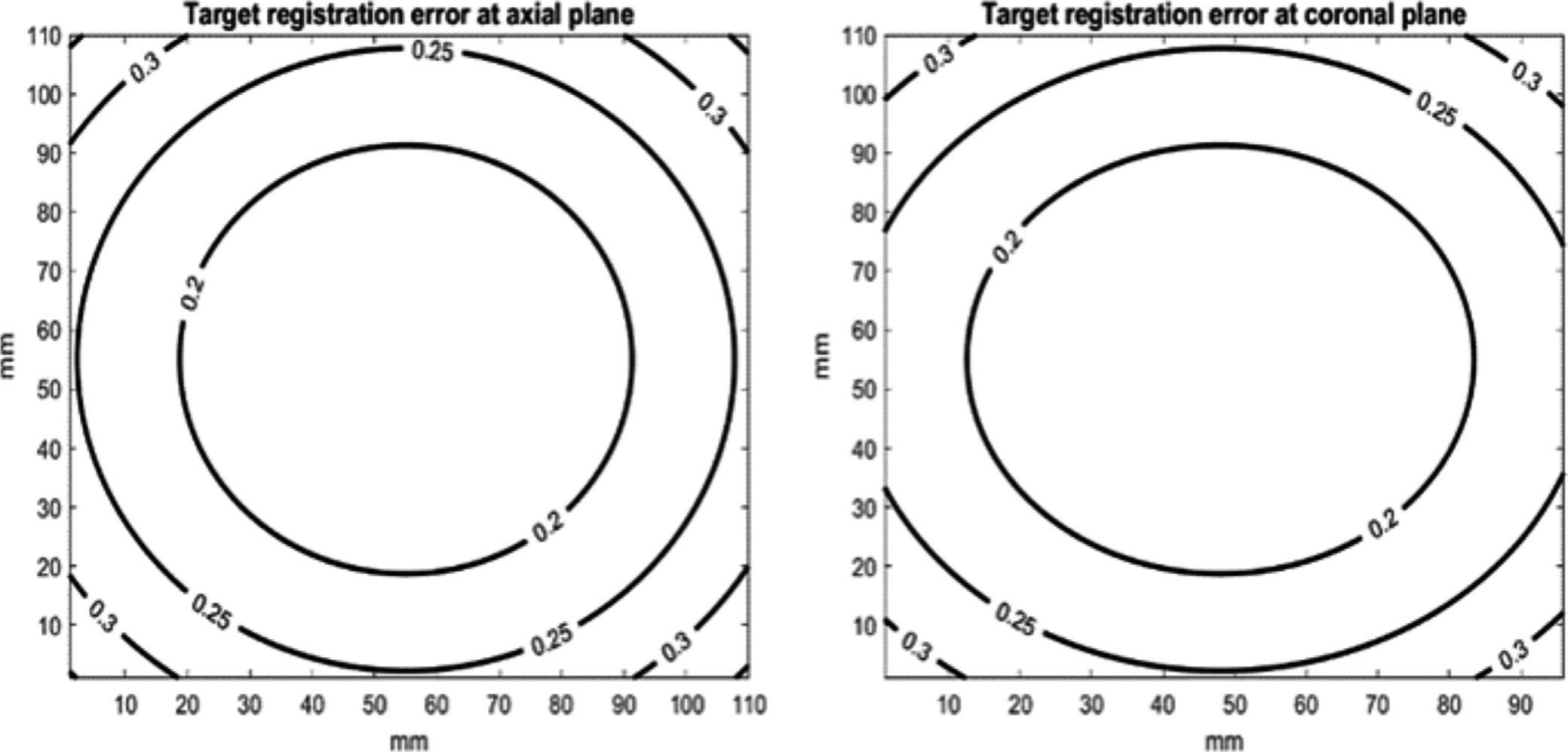
Estimated TRE shown in axial and coronal plane.

Additionally, the prominence values of the fiducial and other points are shown in the histogram (Fig. 15). The top two histograms comprise data from CT images and the two histograms at the bottom comprise data from OCT images. First, the prominence values of the fiducial markers on CT images are higher than what on OCT images. Moreover, prominence values at other pixels are much smaller than the fiducial pixels in both image sets, which leads to the negligible possibility of misdetection of the fiducial points. In CT images, prominence values are in the range of 0 to 500 at non‐fiducial pixels and 8000 to 19 000 at fiducial pixels. In OCT images, prominence values are in the range of 0 to 16 at non‐ fiducial pixels and 4150 to 4510 at fiducial pixels. From the analysis of 20 fiducial registrations, FRE was measured to be 0.62 mm and FLE was calculated to be 0.44 mm using Eq. (4). TRE in the 3D space can be estimated from FLE by using Eq. (5). Figure 16 shows the isovalue lines of TRE in the axial and coronal views. Due to symmetric configuration of the fiducial points, the results in coronal view are the same as those in sagittal view. In the region of measured dose distribution, which is usually at the center of the dosimeter, the estimated TRE is smaller than 0.3 mm. In addition, TRE was estimated by analyzing 10 fiducial markers, previously registered as targets. After registration, all of them are shown to be at the same coordinates in the CT and OCT images. We were unable to measure submillimeter registration errors because the resolution limit of treatment planning exported dose images and CT images is 1 mm.

## 4 DISCUSSION

A robust methodology of 3D IMRT QA using point‐based registration and structure mapping was proposed in this study, which aims to improve the correlation between IMRT QA evaluation and the underlying planning and delivery errors. In previous publications,^5^, ^6^, ^7^, ^8^ concerns were raised about 2D measurement‐based IMRT QA using the gamma index. First of all, investigators presented situations where the conventional single field 2D dose distribution comparison using the gamma index was insensitive in detecting dose errors as well as specific delivery errors due to the relatively low resolution of the dosimeters, loose constraints or errors washed out in the composite dose images. Second, clinical acceptability does not correlate with the passing rate of the gamma index. The gamma failure points could be distributed throughout either the target or critical organs. To have a comprehensive view of an individual treatment plan, the IMRT QA method needs to have the capability of detecting small delivery errors as well as providing spatial information of the errors relative to the important structures. For the cases presented in this study, conventional 2D IMRT QA cannot provide failing points location relative to the structure but only evaluate the overall dose difference. In the first case, ArcCHECK (Sun Nuclear) shows average 97.4% passing rate using 3%, 3 mm criteria. However, this study shows that most of the failing points are at the edge of PTV where dose gradient is high, which influences the PTV coverage. In the second case, although average passing rate using conventional 2D gamma (3%, 3 mm) is only 94.7%, this study shows that the dose coverage of all PTVs is preserved and most of the failing points are at the intermediate dose regions (50–70%) between the two close lesions.

The proposed method can be an effective tool for commissioning of novel treatment techniques, such as multiple lesions radiosurgery treatments. It has sufficient resolution and signal‐to‐noise ratio to detect small delivery errors. Besides, accurate point‐based registration was employed to correlate the measurement coordinate system and planning coordinate system. Accurate registration of the planning and measurement systems enabled acquisition and translation of relevant structural information. Using the proposed method, clinical‐relevant information such as DVH, 3D location of gamma failing points and 2D dose distribution in the high gradient region can be employed to make comprehensive clinical judgments.

The sensitivity and specificity of an IMRT QA method to detect planning or delivery errors relates to the uncertainties of the whole QA procedure. Therefore, the source and magnitude of the uncertainties should be estimated. More significant uncertainties than the errors to be detected could result in a high rate of false positives. The sources of uncertainties of the proposed IMRT QA method include fiducial registration, dose measurements, structures mapping and dosimeter setup. Using the pixel‐to‐pixel mapping of the Eclipse treatment planning system, the uncertainty from structure mapping is negligible. The dosimeter setup error relates to the laser error and operator error, which is similar to all the measurement‐based IMRT QA methods. In this study, errors from fiducial registration were analyzed. FLE, FRE, and TRE were estimated to be less than a millimeter. TRE of pixels in 3D space of the dosimeter was calculated to be smaller than 0.3 mm. The highest resolution of Eclipse treatment planning dose calculation is 1 mm. Therefore, the proposed fiducial markers and configuration can provide sufficient accuracy for dose comparison.

The PRESAGE dosimeter is accurate in terms of relative dose distribution measurement but is not ready for absolute dosimetry. The selection of the normalization point of the measured dose distribution could affect the interpretation of the results. In this study, the normalization point was chosen to be in a uniform high dose region. Moreover there are differences between the inhomogeneity of the real patient and the dosimeter, and thus the magnitude of the discrepancy between the measurement and the calculation evaluated using the phantom could be different than the real discrepancy in the patients. This is the same as all the other measurement‐based IMRT QA methods used routinely in clinical practice. To improve the correlation, phantom size and shape should be close to patient’s geometry. As 3D printing becomes more common and low‐cost, patient‐specific phantom could be utilized for radiotherapy dosimetry.^27^

This work has provided a clinically feasible methodology utilizing an automatic fiducial registration algorithm and commercially available structure‐mapping application in clinical TPS, which is a step toward the implementation of a foolproof 3D dosimetric verification system with organ‐specific dose information for routine clinical use. With the acquired information, organ‐specific dose difference criterion could be implemented in the future. Moreover our study adds on to the current methods for 3D dosimetric analysis by reporting the registration error as part of the dose comparison error. More convenient, user‐independent and time‐efficient optical scanners and programs are being developed at our lab so that 3D dosimetry can become clinically available and easily accessible in the future.

## 5 CONCLUSIONS

In this study, we introduced a robust methodology of 3D measurement‐based IMRT QA for organ‐specific dose comparison. With accurate point‐based registration between measured and calculated image spaces, a precise spatial correlation between the two can be found. In addition, the patient’s anatomical structures can be mapped to the CT images of the phantom using the coordinates of the fiducials. This work demonstrates two clinical cases and shows the capability of 3D organ‐specific dose comparison. In addition, a comprehensive analysis of the registration uncertainties was performed. This work aims to improve the current 2D measurement based IMRT QA and shows the clinical feasibility of 3D dosimetry for future use.

## CONFLICT OF INTEREST

The authors declare no conflict of interest.

## AUTHOR CONTRIBUTIONS

Yi‐Fang Wang^:^ Contribution Statement: The author made substantial contribution to conception and design, drafting the article, analysis, interpretation of data, and final approval of the version to be submitted. Olga Dona: Contribution Statement: The author participated in acquisition of data and drafting the article or revising it critically for important intellectual content and final approval of the version to be submitted. Yuanguang Xu: Contribution Statement: The author participated in drafting the article or revising it critically for important intellectual content and final approval of the version to be submitted. John Adamovics: Contribution Statement: The author participated in drafting the article or revising it critically for important intellectual content and final approval of the version to be submitted. Cheng‐Shie Wuu: Contribution Statement: The author made substantial contribution to conception and design, drafting the article, analysis, interpretation of data, and final approval of the version to be submitted.
